# pH-Dependent Photoinduced Interconversion of Furocoumaric and Furocoumarinic Acids

**DOI:** 10.3390/molecules26092800

**Published:** 2021-05-10

**Authors:** Vladislav V. Skarga, Anton A. Matrosov, Artemiy I. Nichugovskiy, Vadim V. Negrebetsky, Mikhail A. Maslov, Ivan A. Boldyrev, Mikhail V. Malakhov

**Affiliations:** 1 Institute of Translational Medicine, Pirogov Russian National Research Medical University, 1 Ostrovityanov Str., 117997 Moscow, Russia; skargavlad@gmail.com (V.V.S.); fmpfan@rambler.ru (A.A.M.); nmr_rsmu@yahoo.com (V.V.N.); ivan@lipids.ibch.ru (I.A.B.); 2 Lomonosov Institute of Fine Chemical Technologies, MIREA—Russian Technological University, 86 Vernadsky Ave., 119571 Moscow, Russia; ashpwnz77@gmail.com (A.I.N.); mamaslov@mail.ru (M.A.M.); 3 Shemyakin-Ovchinnikov Institute of Bioorganic Chemistry, Russian Academy of Sciences, 16/10 Miklukho-Maklaya Str., 117997 Moscow, Russia

**Keywords:** furocoumaric acid, furocoumarinic acid, (*Z* → *E*)-photoisomerization, (*E* → *Z*)-photoisomerization, UV irradiation, pH dependence, fluorescence, photoswitch, quantum chemistry calculations, psoralens, furocoumarins, photoproducts

## Abstract

Photo-controlled or photo-regulated molecules, especially biologically active and operating in physiological conditions, are in steady demand. Herein, furocoumaric and furocoumarinic acids being (*Z/E*)-isomers relative to each other were obtained in two stages starting from psoralen: the alkaline solvolysis of psoralen led to furocoumaric acid, which was further *Z* → *E* photoisomerized (365 nm) to furocoumarinic acid. The kinetics of *Z* → *E* photoisomerization was monitored by HPLC and UV-vis spectrophotometry. Photophysical characteristics in the aqueous phase for both acids, as well as the reversibility of (*Z*/*E*) photoisomerization process, were also assessed. Furocoumarinic acid was found to be visibly fluorescent at pH 2.0–12.0, with the maxima of fluorescence emission spectra being pH-dependent. The reverse *E* → *Z* photoisomerization predicted by quantum chemistry calculations as energetically favorable for the monoanionic form of furocoumarinic acid was proved in the experiment while being complicated by pyrone ring closure back to psoralen in acidic and neutral conditions. The preparative synthesis of furocoumarinic acid outlined in this work is particularly valuable in view of a wide range of pharmacological effects previously predicted for this compound.

## 1. Introduction

Psoralens are plant-derived or synthetic linear furanocoumarins, which are commonly used as medicinal UVA-photosensitizers [1,2]. Current psoralen-based phototherapies (PUVA-therapy and photopheresis) are versatile, cost-efficient, and safe protocols widely used for the treatment of various T-cell mediated and hyperproliferative skin disorders [3,4,5,6]. These therapies are based on antiproliferative, immunosuppressive, and apoptotic effects toward keratinocytes and immune cells, thus being considered as photochemotherapy and photoimmunotherapy [4,6,7,8,9]. Anti-tumor, anti-inflammatory, and antibacterial effects of psoralens were also reported [10,11,12], as well as their effects on cell cycle, apoptosis, and differentiation [13].

Photolysis of psoralens in vitro provides the complex mixtures of biologically active photoproducts comprehensively reviewed by S. Caffieri [14]. Photoproducts of psoralens were found to induce phototoxic and dark membranolytic effects and apoptosis, as well as affect cell proliferation and differentiation [13,14,15,16,17,18,19,20]. More importantly, photoproducts of psoralen photooxidation were able to induce the immunotherapeutic effect in vivo [20,21,22] and selectively induce apoptosis in vitro in the transformed T-cell lines (*Jurkat* cell line) [17], suggesting the crucial role of these photoproducts in the realization of the immunotherapeutic effects of psoralen-based phototherapies.

**Scheme 1:**
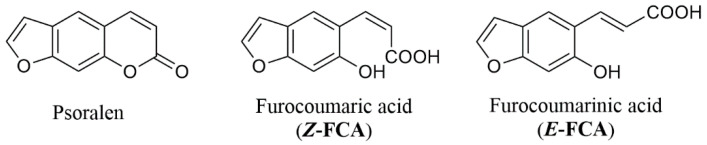
Structure of the subject compounds.

Furocoumaric acid [(*Z*)-3-(6-hydroxybenzofuran-5-yl)acrylic acid; *Z*-FCA] is an α-pyrone ring-opening (photo)product of psoralen, while furocoumarinic acid [(*E*)-3-(6-hydroxybenzofuran-5-yl)acrylic acid; *E*-FCA] is *E*-isomer of *Z*-FCA (Scheme 1). Recently, a wide range of pharmacological effects was predicted for *E*-FCA by means of chemoinformatic analysis [23]. These effects include a number of protective (i.e., neuro-, vaso-, and radioprotective) effects, antimutagenic, anticarcinogenic, antioxidant, and anti-inflammatory activities, and even a prospective dermatologic (in particular, antipsoriatic) therapeutic activity.

Structurally similar acids and their O-methylated analogs were previously reported for 8-methoxypsoralen (8-MOP) and 5-methoxypsoralen [24,25,26,27]. These compounds have been construed as water-soluble precursors (“pre-psoralens”), yielding the corresponding psoralens in situ in the course of photocyclization reaction. It was found that this reaction proceeds at physiological pH and temperature through the stage of ***E*→ *Z*** photoisomerization of the acid followed by the lactonization process [25,26,27].

Reversibly photoisomerizable (*Z*/*E*)-FCAs might be considered as a cinnamate light-activated molecular switch (photoswitch). Photoswitches and molecular motors based on photo-switchable molecules have found application in a number of areas, including biomolecular and analytical chemistry, as well as material sciences [28,29,30]. Photochemical control of material functions using cinnamate photoswitches was reported for hydro/nanogels, micelle solutions, and polymer networks [31,32,33,34]. *o*-Hydroxycinnamate phototriggers were used for image-guided photo-regulated uncaging of biologically relevant and cosmeceutical agents [35,36].

Taking into consideration the prospective pharmacological activities of (*E*)-FCA and the practical relevance of its photophysical properties, this work was aimed to prepare (*Z*/*E*)-FCAs using previously described protocol [24,25,26,27] and to assess their photophysical behavior in the aqueous phase and reversibility of (*Z*/*E*) photoisomerization process.

## 2. Results and Discussion

### 2.1. Psoralen Pyrone Ring-Opening

α-Pyrone ring-opening under alkaline conditions are well-known reactions [37,38], and derivatives of 8‑MOP and their O-methylated analogs were obtained earlier using this approach [24,25,26,27].

**Figure 1:**
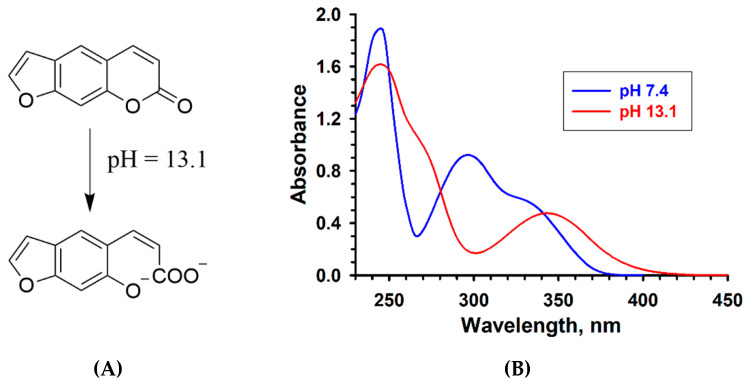
Psoralen pyrone ring-opening. (**A**) Reaction scheme. (**B**) UV-vis absorption spectra of 0.1 mM psoralen solution in PBS (pH 7.4, 1% of ethanol) and after alkalizing to pH 13.1.

Psoralen pyrone ring-opening after treatment with 1 M aq. NaOH was accompanied by significant changes in the UV‑vis absorption spectra (Figure 1). An absorption spectrum of psoralen in PBS (pH 7.4, 1% of ethanol) was characterized with two absorption maxima at 245 and 296 nm and no absorption above 400 nm. After the psoralen solution was alkalized (pH 13.1), the maximum at 296 nm disappeared, and a new broad absorption band with a maximum at 343 nm was formed. A weak absorption in the visible region (up to 430 nm) also appeared. Earlier, similar spectral changes were observed for 8‑MOP ring-opening products in the alkaline water/ethanol mixture (95/5, pH 12) [24]. These similarities with 8‑MOP suggest that strong alkalizing of psoralen solution also results in pyrone ring-opening yielding *Z*-FCA dianion.

Preparative isolation and characterization of *Z*-FCA were not performed since these processes are complicated due to the well-studied process of pyrone ring back‑closure in neutral and acidic conditions previously noted for 8-MOP [25,26,27].

### 2.2. Z→ E Photoisomerization

According to earlier findings, UVA irradiation of the dianionic 8‑MOP ring-opening product in alkaline conditions results in its *Z* → *E* photoisomerization, and highly diluted solutions, as well as the absence of oxygen, are needed to avoid dimerization and/or photooxidation processes [24,25,26,27]. *Z* → *E* photoisomerization of psoralen ring-opening product (*Z*-FCA dianion) at pH 13.1 was performed using increasing fluences of UVA irradiation (365 nm, 0–252 kJ m^−2^) and monitored using reverse-phase HPLC and UV-vis absorption spectroscopy.

In order to make the samples suitable for HPLC analysis, the obtained alkaline UVA-irradiated solutions were acidified to pH 2 with 1 M aq. HCl prior to analysis. HPLC analysis of the acidified non-irradiated sample provides a picture of a single peak with the retention time (R_t_) of 14.3 min, which corresponds to R_t_ of psoralen (Figure S1). Thus, the acidification of the initial non-irradiated sample leads to complete *Z*-FCA pyrone ring back‑closure to psoralen similarly to 8-MOP [25,26,27], and the HPLC peak area for psoralen may serve as a quantitative measure of *Z*-FCA remained after UVA irradiation.

**Figure 2:**
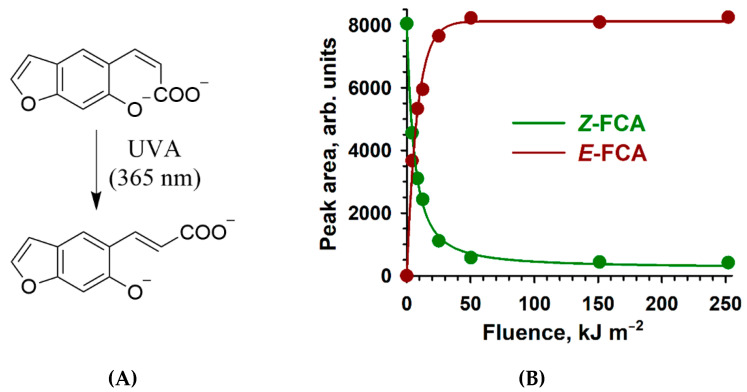
Fluence-dependent HPLC changes during the *Z* → *E* photoisomerization process. (**A**) Reaction scheme. (**B**) *Z*-FCA and *E*-FCA peak areas as monitored by HPLC.

Fluence-dependent HPLC changes during the *Z* → *E* photoisomerization process are summarized in Figure 2. At the minimal UVA fluence (4.2 kJ m^−2^), HPLC analysis showed about a half-decrease in *Z*-FCA in solution, and the second peak with R_t_ = 13.1 min, which may be attributed to fully protonated *E*-FCA was also detected (Figure S1). Further increase in UVA fluences (up to 50.4 kJ m^−2^) resulted in the maximal consumption of *Z*-FCA (of ~95%), and the peak of *E*-FCA reached the plateau. At higher UVA fluences (50.4–252 kJ m^−2^), no valuable changes for both *Z*-FCA and *E*-FCA amounts were found on chromatograms. In addition, no other peaks comparable with those of *Z*-FCA and *E*-FCA were found even at the highest UVA fluence (252 kJ m^−2^), which corresponded to 5 min of irradiation using a high radiant flux UVA source (Figure S1). This suggests that no other sufficient photoinduced or post-irradiation processes occur during or after *Z* → *E* photoisomerization and this approach seems applicable for the preparative synthesis of *E*-FCA.

**Figure 3:**
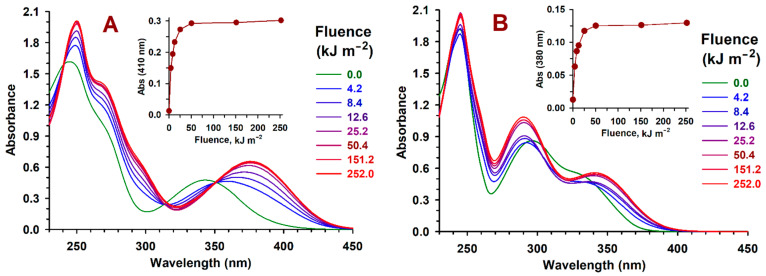
UV-vis absorption changes upon ***Z*→ *E*** photoisomerization process. Conditions: (**A**) spectral changes immediately after irradiation of the alkaline solution of psoralen (0.1 mM, 1% of ethanol, pH 13.1) with increasing fluences of UVA irradiation (365 nm, 0–252 kJ m^−2^). The insert shows the photometric changes at 410 nm; (**B**) spectral changes after strong acidification of the irradiated samples to pH 2. The insert shows the photometric changes at 380 nm.

Absorption changes during the *Z* → *E* photoisomerization process in alkaline solutions were also fluence‑dependent (Figure 3A). After minimal UVA irradiation (4.2 kJ m^−2^), the *Z*-FCA dianion absorption maxima at 245 and 343 nm were shifted to 249 and 360 nm, respectively. Upon gradual increase in UVA fluences up to 50.4 kJ m^−2^, the absorption at 249 nm was also increased with a small shift of maximum to 250 nm, while the red shift of the rising 360 nm peak was more pronounced (to 376 nm). At higher fluences, no valuable changes of absorption or peak positions were found. The presence of isobestic points at 240, 314, and 350 nm further implies the *Z*-FCA dianion conversion to *E*-FCA dianion.

Spectral changes after acidification of the irradiated samples to pH 2 were less prominent (Figure 3B). The absorption spectrum of acidified non-irradiated solution coincided with the psoralen absorption spectrum (Figure 1), closely corresponding to HPLC analysis data (Figure S1). At low fluences (4.2–12.6 kJ m^−2^), the acidified irradiated solutions consist of the newly formed product (presumably *E*-FCA) and psoralen, the latter being the product of the pyrone ring back-closure of non-isomerized *Z*-FCA. Thus, upon an increase in UVA fluences, the absorption spectrum shifted gradually to the spectrum of *E*-FCA through the isobestic points at 304 and 332 nm. At higher fluences (equal or above 25.2 kJ m^−2^), the quantities of psoralen were too small to contribute significantly to the absorption of solutions, and the absorption spectra might be attributed to the spectrum of fully protonated *E*-FCA with characteristic maxima at 245, 290, and 342 nm.

Fluence-dependent photometric changes both in alkaline and acidified irradiated solutions (Figure 3A,B, inserts) are in close agreement with HPLC analysis data (Figure 2). Thus, *Z* → *E* photoisomerization process may be monitored by routine photometric data acquisition.

The above-described *Z* → *E* photoisomerization approach with photometric *Z* → *E* conversion monitoring was scaled up for preparative synthesis of *E*-FCA (see Section 3.4). It was found that UVA‑irradiation of 0.6 L sample for 9 min was aligned with maximal absorbance changes at 410 nm (Figure S2). According to HPLC analysis, this time point matches with 93% conversion of *Z*-FCA to *E*-FCA. The isolated and purified *E*-FCA was characterized using NMR and MS methods. The recorded ^1^H and ^13^C-NMR spectra of *E*-FCA are presented in Figures S3 and S4. The coupling constant values for α,β vinyl protons (both of about 16 Hz) imply the *E* character of this bond. Similar observations were made earlier for the methoxy derivative of *E*-FCA prepared from 8-MOP [24,25,26,27]. HRMS spectra recorded both in positive and negative modes are presented in Figures S5 and S6, and the values obtained for [M+H]^+^ and [M−H]^–^ correspond to calculated values.

### 2.3. pH-Dependent Absorption and Fluorescence Characteristics of (Z/E)-FCAs

Both *Z*-FCA and *E*-FCA have acidic dissociable protons at the carboxyl and phenolic groups. Thus, depending on pH, these acids may present in fully protonated, monoanionic, and dianionic forms, as well as mixtures thereof.

However, a process of pyrone ring back‑closure to psoralen in neutral and acidic conditions previously noted for methoxy derivative of *Z*-FCA [25,26,27] and confirmed for *Z*-FCA in the present work (see Section 2.2. above) governs the presence of *Z*-FCA in protic solvents as a dianionic form at the alkaline pH range only.

Theoretical pH-dependent relative distribution of fully protonated and ionized forms of *E*-FCA was calculated using the Marvin pKa plugin (Figure S7). The acid-base dissociation constants for *E*-FCA in aqueous solutions were predicted as being pK_a1_ = 3.61 (for the carboxyl proton dissociation) and pK_a2_ = 8.43 (for the phenolic proton dissociation). In addition, it was calculated that a fully protonated form of *E*-FCA is predominant at pH < 2.0, with monoanionic and dianionic forms being predominant around pH 6.0 and at pH > 10, respectively.

**Figure 4:**
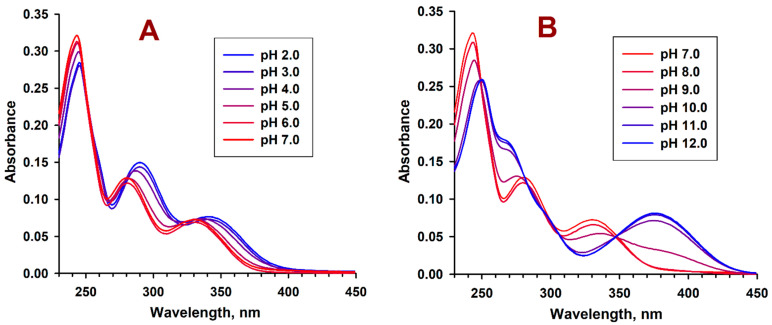
UV-vis absorption changes upon pH titration of the aqueous *E*-FCA solution (12.5 µM, 0.5% of ethanol). Conditions: (**A**) absorbance changes upon neutral to acidic titration of *E*-FCA with 1 M aq. HCl; (**B**) absorbance changes upon neutral-to-alkaline titration of *E*-FCA with 1 M aq. NaOH.

UV-vis absorption changes upon pH titration of the aqueous *E*-FCA solution in the range of pH 2.0–12.0 are presented in Figure 4. The absorption spectrum of *E*-FCA at pH 2.0 was characterized with three absorption maxima at 245, 290, and 342 nm. Upon increase in pH values, all the maxima showed the hypsochromic shifted to 243, 280, and 330 nm, respectively, with the minor changes in absorbance values (Figure 4A). The most vivid changes at pH 6.5~7.0 can be attributed to the complete dissociation of the carboxylic protons. Further increase in pH values (to alkaline region) was accompanied by the dramatic changes of the absorption spectra, which resulted from the dissociation of the phenolic protons. At the pH > 8.0, the absorption spectra underwent the vivid transformations (Figure 4B): the maximum at 243 nm was shifted to 250 nm, and two other maxima at 280 and 330 nm were transformed to a shoulder at ~270 nm and a new broad maximum at 376 nm (i.e., with a bathochromic shift of 46 nm) with a prominent absorption in the visible region. According to the obtained absorption spectra, the predicted pK_a1_ and pK_a2_ values should be corrected to about 4.5 and 9.2, respectively, especially considering the fact that the close values were previously reported for the methoxy derivative of *Z*-FCA [24]. In addition, the absence of significant changes in absorbances at pH < 3.0 may be indicative of the pH range with the predominance of fully protonated *E*-FCA.

**Figure 5:**
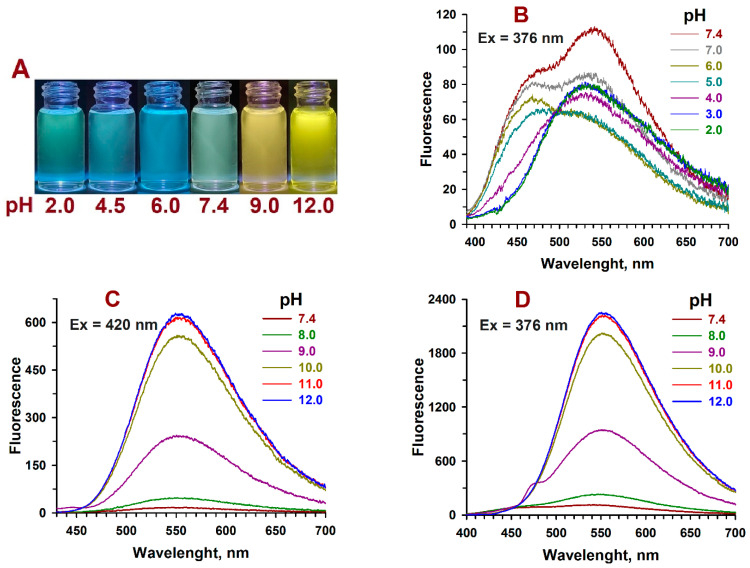
Fluorescence changes upon pH titration of the aqueous *E*-FCA solution (12.5 µM, 0.5% of ethanol). Fluorescence images of the aqueous *E*-FCA solution at various pH values under UV LED light (365 nm) (**A**); fluorescence changes upon neutral to acidic titration of *E*-FCA with 1 M aq. HCl under excitation at 376 nm (**B**); fluorescence changes upon neutral-to-alkaline titration of *E*-FCA with 1 M aq. NaOH under excitation at 420 nm (**C**) or 376 nm (**D**).

Fluorescence changes upon pH titration of *E*-FCA were also pH-dependent (Figure 5) and visible to the naked eye (Figure 5A). Two wavelengths, i.e., 376 and 420 nm, were chosen for excitation of the aqueous *E*-FCA solution in the range of pH 2.0–12.0: the first one is the “universal” excitation wavelength for all three forms of *E*-FCA, while the second one is specific for the dianionic form of *E*-FCA.

The fluorescence emission spectra of *E*-FCA at pH 2.0 and pH 3.0 had equal intensities and a peak at 536 nm (Figure 5B). Increasing the pH value to 4.0–6.0 resulted in a hypsochromic shift of the emission maximum to ~470 nm with the transformation of the peak at 536 nm to a shoulder. Further increase in pH value in the range of 6.0–7.4 was accompanied by a slight increase in fluorescence intensity at ~470 nm and the appearance of another peak at ~540 nm. The fluorescence intensity of the latter notably increased in the range of 6.0–7.4, and finally, this peak became predominant at pH 7.4 with the emission maximum at 543 nm. All these transformations of the emission spectra at the acidic-to-neutral pH range might indicate the complete dissociation of the carboxylic protons of *E*-FCA, while the new rising peak at pH 6.0–7.4 can be attributed to the initial stages of dianion formation. These features are in suitable agreement with absorption changes upon pH titration presented in Figure 4A. Moreover, the strong contribution of dianionic form in the fluorescence intensity at pH 6.0–7.4 suggests that this form is more fluorescent than the others.

The fluorescence intensity measurements at the neutral-to-alkaline pH range finalized the peak value for the dianionic form of *E*-FCA (Figure 5C,D). Under excitation at 420 nm, the fluorescence emission maximum for the dianionic form of *E*-FCA was found to be equal to 555 nm (Figure 5C). Meanwhile, at pH values in the range of 7.4–9.0, the contribution of other forms is still visible, especially under excitation at 376 nm where all three forms of *E*-FCA absorb (Figure 5D). These features are also in suitable agreement with absorption changes upon pH titration presented in Figure 4B. Furthermore, the vivid amplification of fluorescence intensity for the dianionic form of *E*-FCA needs to be noted.

The fluorescence quantum yields (*Φ*) of *E*-FCA at various pH were measured relative to quinine sulfate in 0.5 M H_2_SO_4_ (*Φ* = 54.6%) used as a standard (Figure S8). The calculated data confirmed our prediction, which was made in the course of spectral studies: the *Φ* of dianionic form of *E*-FCA at pH 12.0 was 7-fold higher than that of the fully protonated form (2.1% vs. 0.3%, respectively). Moreover, the *Φ* values calculated for *E*-FCA at pH 6.0 and 7.4 (1.6% vs. 2.0%, respectively) were found to be remarkably close to the *Φ* value of the dianionic form of *E*-FCA. Combined with fluorescence spectra presented in Figure 5B, these data argue for the complex character of *E*-FCA solutions at neutral pH region, which presumably contains the monoanionic form of *E*-FCA with admixtures of fully protonated and dianionic forms, the latter strongly contributing to fluorescence intensity.

pH-Dependent photophysical characteristics of (*Z/E*)-FCAs described above were summarized below in Table 1, being added with absorption data for *E*-FCA in ethanol (Figure S9) and photophysical data for methoxy derivative of *E*-FCA [24].

The photophysical stability of *E*-FCA in different pH regions was confirmed by fluorescence measurements during cyclic changing of pH values between pH 2.0 and 12.0. Monitoring of fluorescence emission under excitation at 376 and 420 nm at these pH values revealed cyclic recovery of emission intensities without any significant changes (Figure S10).

### 2.4. Computational Studies

**Figure 6:**
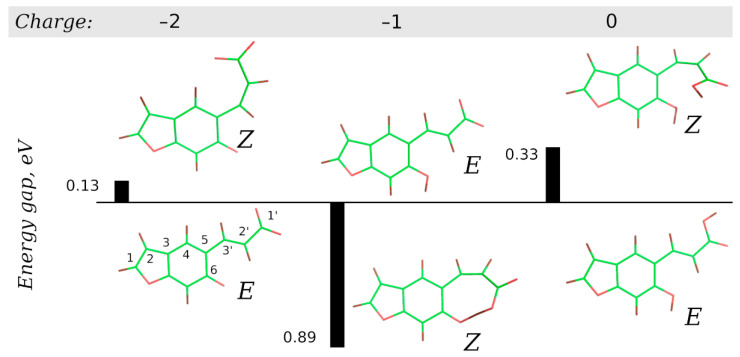
Optimized geometries for the dianionic, monoanionic, and protonated forms of (*Z/E*)-FCAs (with charges of −2, −1, and 0, respectively). Bars represent energy gaps (eV) between corresponding forms of *E*-FCA and *Z*-FCA.

Quantum chemistry calculations were used to obtain optimized geometries and corresponding energy gaps for the protonated, monoanionic, and dianionic forms of *E*-FCA and *Z*-FCA (Figure 6). Molecular orbitals for all forms of (*Z*/*E*)-FCAs are shown in Figure S11.

The calculated energies for dianionic and protonated forms of *Z*-FCA were found to be higher than the energies of corresponding forms of *E*-FCA. Thus, *Z* → *E* isomerization is energetically favorable for these forms. In contrast, the energy of monoanionic *E*-FCA is lower than the energy of monoanionic *Z*-FCA, and the reverse process of *E → Z* isomerization becomes energetically favorable.

The values of dihedrals, angles, and bond lengths calculated for (*Z/E*)-FCAs are presented in Table 2. It can be noticed that the spatial structure of *E*-FCA does not change sufficiently during the loss or addition of a proton (Figure 6). The molecule is almost planar: the 5-3′-2′-1′ and 6-5-3′-2′ dihedral values are close to 180° and 0°, respectively. The length of the 3′-2′ (double) and 5-3′ (single) bonds are also conventional for conjugated systems.

In contrast, the geometries of protonated and both ionized forms of *Z*-FCA are notably different (Figure 6). The protonated and monoanionic forms of *Z*-FCA are not planar, as the 5-3′-2′-1′ and especially the 6-5-3′-2′ dihedral values are far from 0° and 180°, respectively. In the case of protonated form, a deviation from planarity is bigger, and the 6-5-3′-2′ dihedral value is about 43°. Moreover, the large degree of deviation from planarity also affects the double bond conjugation in the protonated *Z*-FCA. The 3′-2′ (double) bond shortens to 1.338 Å, while the 5-3′ (single) bond elongates to 1.470 Å (i.e., the degree of bond conjugation decreases).

The dianionic form of *Z*-FCA appeared to be planar, since calculated the 5-3′-2′-1′ and the 6-5-3′-2′ dihedral values are close to 0° and 180°, respectively.

Furthermore, a sufficient deviation of bond angles from conventional values (of 120° for sp2 hybridization) was revealed for all forms of *Z*-FCA, as well as the formation of the internal hydrogen bond in the monoanionic form of *Z*-FCA.

### 2.5. E → Z Photoisomerization

**Figure 7:**
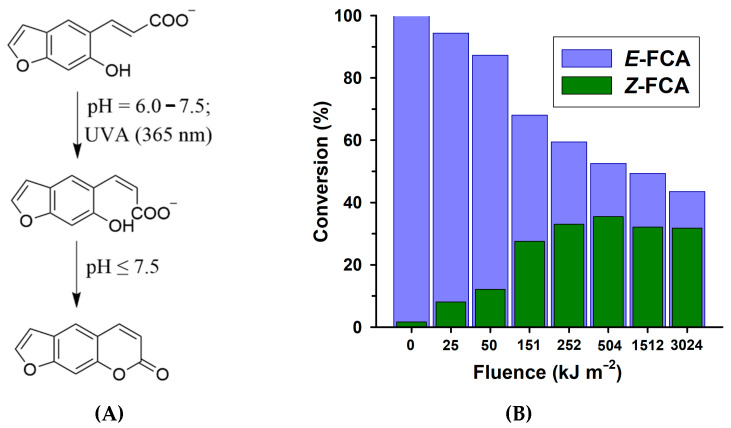
HPLC-controlled fluence-dependent *E* → *Z* conversion in *E*-FCA solutions (0.1 mM, 1% ethanol) UVA-irradiated at pH 6.5. (**A**) Reaction scheme. (**B**) Normalized conversion of *E*-FCA to *Z*-FCA as monitored by HPLC (raw HPLC data are presented in Figure S12).

According to computational studies, the *E* → *Z* photoisomerization process is energetically favorable for the monoanionic form of *E*-FCA. With the proviso that this form of *E*-FCA is predominant at neutral pH region, the *E* → *Z* photoisomerization process was monitored by HPLC analysis of *E*-FCA solutions UVA-irradiated at pH 6.5 (Figure 7).

It was found that non-irradiated *E*-FCA solutions contained ~1.7% of *Z*-FCA as an impurity after synthesis. Upon UVA irradiation, gradual fluence-dependent *E* → *Z* conversion was observed. Similar to *E* → *Z* photoisomerization, HPLC-controlled formation of psoralen during *Z*-FCA pyrone ring back‑closure was used as a measure of *E* → *Z* conversion. At fluences above 151 kJ m^−2^, the process of *E* → *Z* photoisomerization was slowed down. Moreover, at higher fluences (>252 kJ m^−2^), psoralen content reached the plateau (or even slightly decreased), while *E*-FCA content still continuously diminished. This effect might indicate a simultaneous formation of *Z*-FCA accompanied by partial photolysis of psoralen newly formed in the course of *E* → *Z* conversion.

## 3. Materials and Methods

### 3.1. Materials

Psoralen (≥98% purity) was purchased from ChemicalPoint (Deisenhofen, Germany). Other reagents and solvents were purchased from commercial suppliers (Sigma‑Aldrich, St. Louis, MO, USA; Acros Organics, Geel, Belguim; Fisher Scientific, Loughborough, UK; J.T. Baker, Deventer, Netherlands) and used without additional purification. All the organic solvents were of HPLC grade.

Ultra-pure water (18.2 MΩ cm) produced on an OMNI‑Analytic water purification system (Xiamen RSJ Scientific Instruments Co., Xiamen, China) was used for the preparation of all aqueous solutions, as well as for spectral and HPLC analyses. Phosphate buffered saline (PBS, pH 7.4 ± 0,1, Biotechnology grade, VWR Life Science AMRESCO, Solon, OH, USA) was prepared by dissolving the PBS tablets in ultra-pure water. Stock solutions of psoralen and *E*-FCA were prepared in distilled ethanol and stored in the dark.

### 3.2. UVA Irradiation Procedures

UVA irradiation procedures were performed on air at ambient temperature (23 ± 2 °C) with continuous stirring using a magnetic stirrer.

For research studies, the samples were UVA-irradiated in a 5 mL optic glass cuvette (1.0 cm path length) using a high radiant flux UV LED array OTLH-0480-UV (365 nm, full width half maximum = 14.5 nm, Opto Technology Inc.; Wheeling, IL, USA). The UVA fluence rate on the front wall of the cuvette (840 W m^−2^) was measured using Waldmann UV-meter (H. Waldmann GmbH & Co., Villingen-Schwenningen, Germany).

For preparative purposes, a large-volume (0.6 L) sample was UVA-irradiated in a 1 L flat-bottomed glass flask using BIO‑LINK BLX‑365 irradiation system (365 nm, 5 × 8 W UVA tubes, Vilber Lourmat, Collégien, France).

### 3.3. Analytical Procedures

All analytical procedures were performed on air at ambient temperature (23 ± 2 °C).

UV-vis absorption spectra were recorded in a 1.0 cm path length quartz cuvette (Hellma Analytics, Müllheim, Germany) using a Shimadzu UV-1601PC spectrophotometer.

The corrected fluorescence emission spectra were recorded in a 1.0 cm path length quartz cuvette (Hellma Analytics, Müllheim, Germany) using a Hitachi F-7000 spectrofluorometer. Both excitation and emission slits were set at 5 nm. To minimize the reabsorption effect, fluorescence measurements were performed in samples with absorbances not exceeded 0.1 at and above the excitation wavelength. The fluorescence quantum yields (*Φ*) of *E*-FCA were measured relative to quinine sulfate in 0.5 M H_2_SO_4_ (*Φ* = 54.6%) as a standard [39].

Analytical reverse-phase HPLC analyses were performed using an Agilent 1200 DAD‑HPLC system (Agilent Technologies, Santa Clara, CA, USA) in gradient elution mode under conditions indicated in the Electronic Supplementary Information file. Data acquisition and processing were performed using a ChemStation^®^ software system (Agilent Technologies, Waldbronn, Germany).

Both ^1^H-NMR and ^13^C-NMR spectra were recorded on Bruker DPX 300 FT‑NMR spectrometer (at 300 and 75 MHz, respectively) using tetramethylsilane as an internal standard and DMSO‑*d*_6_ as a solvent.

The mass spectra were recorded both in positive and negative modes using an Orbitrap Elite mass spectrometer (Thermo Fisher Scientific, Waltham, MA, USA) equipped with a HESI probe (direct infusion rate = 5 μL min^−1^; spray voltage = 4 kV).

The pH values were measured using a Hanna HI 83,141 pH meter and corrected by the addition of 1 M aq. HCl or 1 M aq. NaOH as appropriate.

### 3.4. Preparation and Characterization of E-FCA

Psoralen (112 mg, 0.602 mmol) was dissolved in 60 mL of EtOH and added to 540 mL of 0.3 M aq. NaOH solution to provide 1 mM alkaline solution of *Z*-FCA containing 10% of EtOH. This solution was transferred to a 1 L flat-bottomed glass flask (~2 cm layer) and UVA-irradiated (365 nm) on air for 9 min with continuous stirring. The resulting solution (0.6 L) was acidified to pH 3.0 by the addition of 1 M aq. HCl, then extracted with ethyl acetate (3 × 80 mL), and the organic phases were washed with water (3 × 50 mL). The combined organic extract was dried over Na_2_SO_4_, filtered, concentrated using rotary vacuum evaporator, and purified by column flash-chromatography on silica gel (Kieselgel 60, 40–63 μm, Merck) eluting with ethyl acetate/petroleum ether (1:5 → 2:1, v/v) to yield *E*-FCA (94 mg, 76.6%). ^1^H-NMR (300 MHz, DMSO‑d_6_, ppm): δ 6.48 (d, *J* = 16.05 Hz, 1H, CHCOO^–^), 6.75 (dd, *J* = 2.23 Hz, 0.93 Hz, 1H, OCHCH), 7.10 (s, 1H, CHCCHCH), 7.71 (s, 1H, OCCHCOH), 7.72 (d, *J* = 2.22 Hz, 1H, OCHCH), 7.78 (d, *J* = 16.07 Hz, 1H, CHCHCOO^–^). ^13^C-NMR (75 MHz, DMSO-d_6_, ppm): δ 98.42, 107.00, 119.81, 119.94, 120.02, 123.22, 136.84, 145.12, 155.39, 156.30, 171.87. HRMS (HESI): m/z calc. for [M+H]^+^ and [M-H]^–^: 205.0501 and 203.0344; found: 205.0496 and 203.0359.

### 3.5. Theoretical Calculations

Calculations were performed for the dianionic, monoanionic, and protonated forms of **(***Z/E*)-FCAs (with charges of −2, −1, and 0, respectively). Geometries of **(***Z/E*)-FCAs were optimized using the Hartree−Fock method with the def2-SVP basis set followed by B3LYP geometry optimization with def2-TZVPP basis set, the latter also providing single point energy. Calculations were carried out using the ORCA software [40].

## 4. Conclusions

pH-Dependent photoinduced interconversion of furocoumaric and furocoumarinic acids was studied in this work. These acids were prepared using an approach previously described for methoxy substituted psoralens [24,25,26,27], i.e., by means of psoralen α-pyrone ring-opening in alkaline conditions to provide furocoumaric acid (*Z*-FCA) followed by *Z* → *E* photoisomerization (365 nm) yielding furocoumarinic acid (*E*-FCA). The *Z* → *E* photoisomerization process was monitored by HPLC and spectrophotometry, and *E*-FCA was obtained in preparatively useful yield. Assuming a wide range of pharmacological effects previously predicted for *E*-FCA by means of chemoinformatic analysis [23], the preparative synthesis of *E*-FCA outlined in this work is particularly valuable.

For both acids, the main photophysical characteristics were assessed. *E*-FCA was found to be visibly fluorescent on the entire pH range studied (pH 2.0–12.0), with the maxima of the emission spectra being pH-dependent. Hence, although not characterized with significant fluorescence quantum yields, *E*-FCA might be considered as an acido-/basochromic fluorophore in a similar manner to some other compounds previously reported [41,42,43].

Based on quantum chemistry calculations, the reverse *E* → *Z* photoisomerization process was proved to be energetically favorable for the monoanionic form of *E*-FCA. Unfortunately, in acidic and neutral conditions, this process readily resulted in pyrone ring closure back to psoralen. Thus, *E*-FCA still might be regarded as a photoisomerizable water-soluble psoralen precursor while decategorized as an intrinsic photoswitch, which is useful at physiological conditions. Instead, photoswitching between *Z*-FCA and *E*-FCA accompanied with psoralen pyrone ring-opening/closure in neutral to basic conditions could be noted. Considering the fact that the direction of photoisomerization is governed by protonation/deprotonation processes, the combined basochromic and photochromic behavior of *Z*/*E*-FCAs render them functional as a dual-responsive system [41,42].

## Figures and Tables

**Table 1:** Photophysical characteristics of (*Z/E*)-FCAs and relative compounds.

Compound	Parameter ^1^	pH 2.0 ^2^	pH 6.0 ^2^	pH 7.4 ^2^	pH 12.0 ^2^	Ethanol
*Z*-FCA	Absorption				245 (15200)	
					343 (4500)	
	Emission				‑ ^3^	
*E*-FCA	Absorption	245 (20700)	243 (25200)	243 (25100)	250 (21200)	245 (25600)
		290 (10900)	280 (9700)	280 (9700)	376 (6700)	286 (11000)
		342 (5500)	330 (5600)	330 (5500)		342 (6600)
	Emission	536 (0.3)	470 (1.6)	543 (2.0)	555 (2.1)	468 (7.0)
Methoxy *E*-FCA ^4^	Absorption	294 (16000) ^4a^		365 (1000) ^4b^	256 (27000) ^4c^	250 (30000)
					378 (7000) ^4c^	289 (14500)
						336 (5200)
	Emission			540 (0.4) ^4d^		530 (3.0)

^1^ Absorption = peak maxima in nm (molar extinction coefficient in (M cm)^−1^); Emission = peak maximum in nm (fluorescence quantum yield in %). ^2^ Conditions: pH 2.0 and pH 6.0 = aqueous solutions acidified with 1 M aq. HCl to the indicated pH values; pH 7.4 = solutions in phosphate buffered saline (PBS); pH 12.0 = aqueous solution basified with 1 M aq. NaOH to the indicated pH value. Concentrations: absorption measurements—0.1 mM, 1% of ethanol; fluorescence measurements—12.5 µM, 0.5% of ethanol. ^3^ Non-fluorescent, upon UVA irradiation readily isomerize to *E*-FCA (see Section 2.2. above). ^4^ Data presented are from [24]. Conditions: ^4a^ = peak value in water/ethanol mixture (95/5) acidified to pH 1.0 with 1 N aq. HCl; ^4b^ = shoulder value in water/ethanol mixture (95/5) at pH 7.0; ^4c^ = peak values in water/ethanol mixture (95/5) basified to pH 12.0 with 0.01 N aq. NaOH; ^4d^ = peak value in water/ethanol mixture (95/5) at pH 7.0.

**Table 2:** The values of dihedrals, angles, and bond lengths calculated for (*Z/E*)-FCAs.

	Dihedrals (°)		Angles (°)			Bond Lengths (Å)	
Compound *(Charge)*	DH 5-3′-2′-1′	DH 6-5-3′-2′	A 4-5-3′	A 5-3′-2′	A 3′-2′-1′	B 5-3′	B 3′-2′
*E*-FCA *(0)*	179.9	0.2	117.3	130.3	123.2	1.457	1.345
*E*-FCA *(-1)*	176.3	2.0	118.0	131.3	122.2	1.464	1.340
*E*-FCA *(-2)*	176.6	2.5	116.8	131.7	122.6	1.464	1.344
*Z*-FCA *(0)*	5.2	43.2	117.8	131.3	129.2	1.470	1.338
*Z*-FCA *(-1)*	9.4	26.8	151.1	138.9	136.0	1.463	1.352
*Z*-FCA *(-2)*	2.3	175.7	124.1	136.9	136.0	1.463	1.355

## Data Availability

The data presented in this study are available in this article.

## Supplementary Materials

The following supporting data are available online: Figures S1–S12 (raw HPLC data; NMR and HRMS spectra; supporting absorption and fluorescence spectral data; HOMO-LUMO molecular orbitals for (*Z*/*E*)-FCAs).

## Sample Availability

Not available.
