# Supplementary material for: pH-Dependent Photoinduced Interconversion of Furocoumaric and Furocoumarinic Acids
Source: Molecules. 2021 May 10;26(9):2800. doi: 10.3390/molecules26092800 (PMC8126128; doi:10.3390/molecules26092800)
Supplement: Supplementary file 1 [file molecules-26-02800-s001.zip › molecules-1186399-supplementary.pdf]

SUPPLEMENTARY MATERIALS

pH-Dependent photoinduced interconversion of furocoumaric and furocoumarinic acids

Vladislav V. Skarga [1,2], Anton A. Matrosov [1], Artemiy I. Nichugovskiy [2], Vadim V. Negrebetsky [1], Mikhail A. Maslov [2], Ivan A. Boldyrev [1,3] and Mikhail V. Malakhov [1,*]

[1] Institute of Translational Medicine, Pirogov Russian National Research Medical University, 1 Ostrovityanov str.; Moscow 117997, Russian Federation; skargavlad@gmail.com (V.V.S.); fmpfan@rambler.ru (A.A.M.); nmr_rsmu@yahoo.com (V.V.N.); ivan@lipids.ibch.ru (I.A.B.); malakhov.mikhail@gmail.com (M.V.M.)

[2] Lomonosov Institute of Fine Chemical Technologies, MIREA – Russian Technological University, 86 Vernadsky ave.; Moscow 119571, Russian Federation; skargavlad@gmail.com (V.V.S.); ashpwnz77@gmail.com (A.I.N.); mamaslov@mail.ru (M.A.M.)

[3] Shemyakin-Ovchinnikov Institute of Bioorganic Chemistry, Russian Academy of Sciences, 16/10 Miklukho-Maklaya str.; 117997 Moscow, Russian Federation; ivan@lipids.ibch.ru (I.A.B.)

* Correspondence: malakhov.mikhail@gmail.com; Tel.: +7-916-815-5258

Content

Figure S1 HPLC control of *Z* → *E* photoisomerization process.

Figure S2 Photometric monitoring of *Z* → *E* conversion during preparative synthesis of *E*-FCA.

Figure S3 ^{1}H NMR spectrum of *E*-FCA.

Figure S4 ^{13}C NMR spectrum of *E*-FCA.

Figure S5 HRMS spectrum of *E*-FCA recorded in positive mode.

Figure S6 HRMS spectrum of *E*-FCA recorded in negative mode.

Figure S7 Calculated pH dependence of mole fractions for fully protonated, monoanionic and dianionic forms of *E*-FCA.

Figure S8 *E*-FCA fluorescence quantum yield measurements.

Figure S9 Absorption spectra of *E*-FCA in several solvents.

Figure S10 Fluorescence intensity recovery during cyclic changing the pH values of *E*-FCA solution (12.5 μM, 0.5% ethanol) between pH 2.0 and 12.0.

Figure S11 HOMO-LUMO molecular orbitals for (Z/*E*)-FCAs.

Figure S12 HPLC control of *E* → *Z* photoisomerization process.

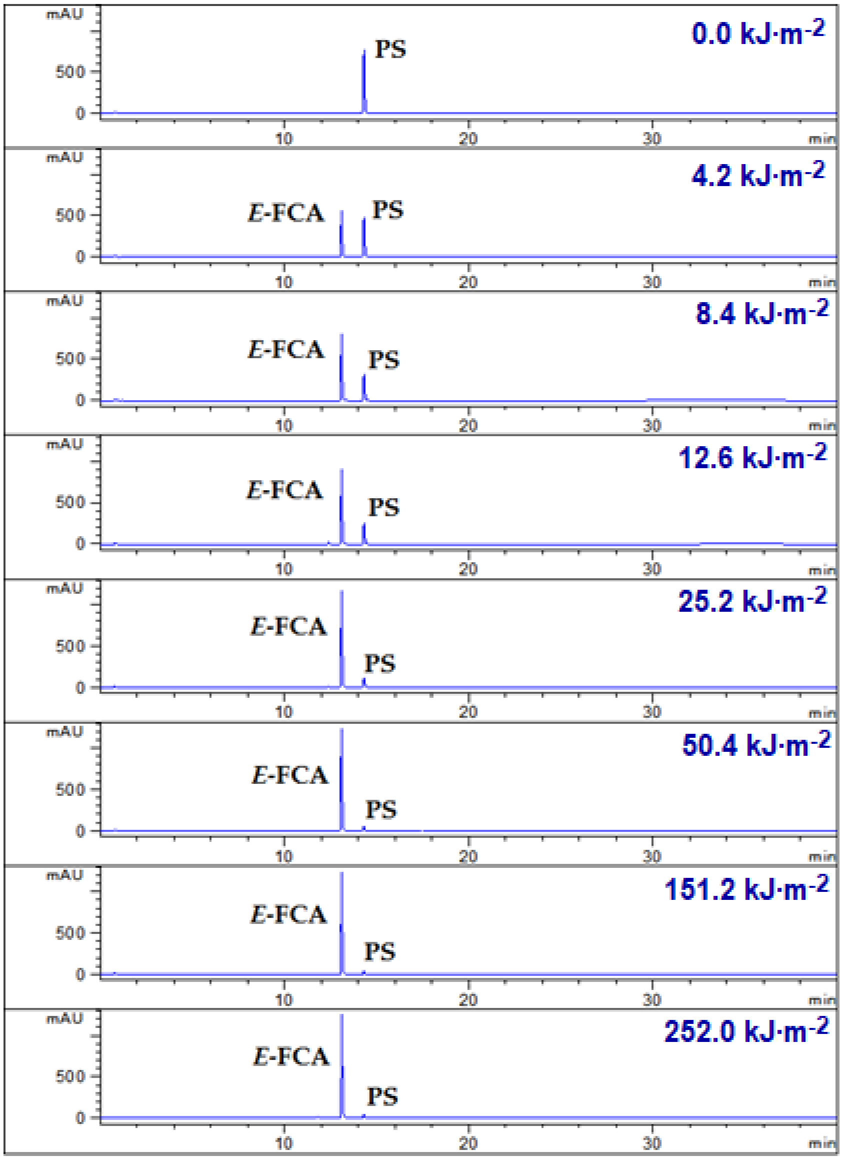

Figure S1 HPLC control of *Z* → *E* photoisomerization process.

UVA irradiated alkaline solutions were acidified to pH 2 with aqueous HCl prior to HPLC analysis.

HPLC analysis conditions: column: Agilent Zorbax® Eclipse XDB C18 Solvent Saver Plus (3.5 μm; 3.0 × 75 mm); mobile phases: A – water + 0.01% TFA, B – acetonitrile + 0.01% TFA; gradient: 2% B for 4 min, 2-50% B over 10 min, 50-70% B over 10 min, 70-100% B over 7 min, 3-min hold at 100% B, 100-2% B over 5 min, 2% B for 1 min; flow rate: 0.5 mL min^{-1}; temperature: 30 °C; injection volume: 30 μL; DAD detection: 246 nm.

UVA fluence: 0 – 252 kJ m^{-2} (top to bottom) corresponding 0 – 5 min of irradiation with high radiant flux UV LED array (365 nm, 840 W m^{-2}).

Top chromatogram corresponds to psoralen (**PS**, R_t = 14.3 min). On other chromatograms, left peak corresponds to fully protonated ***E*-FCA** (R_t = 13.1 min), and right peak corresponds to psoralen as a quantitative measure of Z-FCA remained after UVA irradiation.

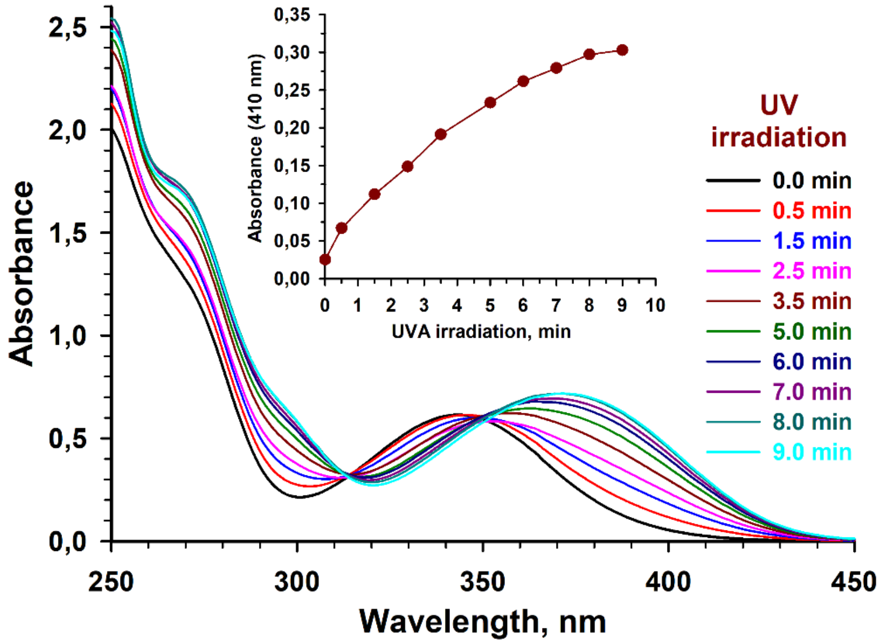

Figure S2 Photometric monitoring of $Z \rightarrow E$ conversion during preparative synthesis of *E*-FCA.

A large-volume sample (0.6 L) of alkaline psoralen solution (1 mM in PBS with 10% ethanol, pH = 13.1) was UVA irradiated (365 nm, 5 × 8 W) in a 1 L flat-bottomed glass flask for 0 – 9 min.

$\boldsymbol{Z \rightarrow E}$ conversion was monitored spectrophotometrically after 10-fold diluting of irradiated sample with PBS. Insert: Kinetics of $Z \rightarrow E$ conversion as monitored at 410 nm.

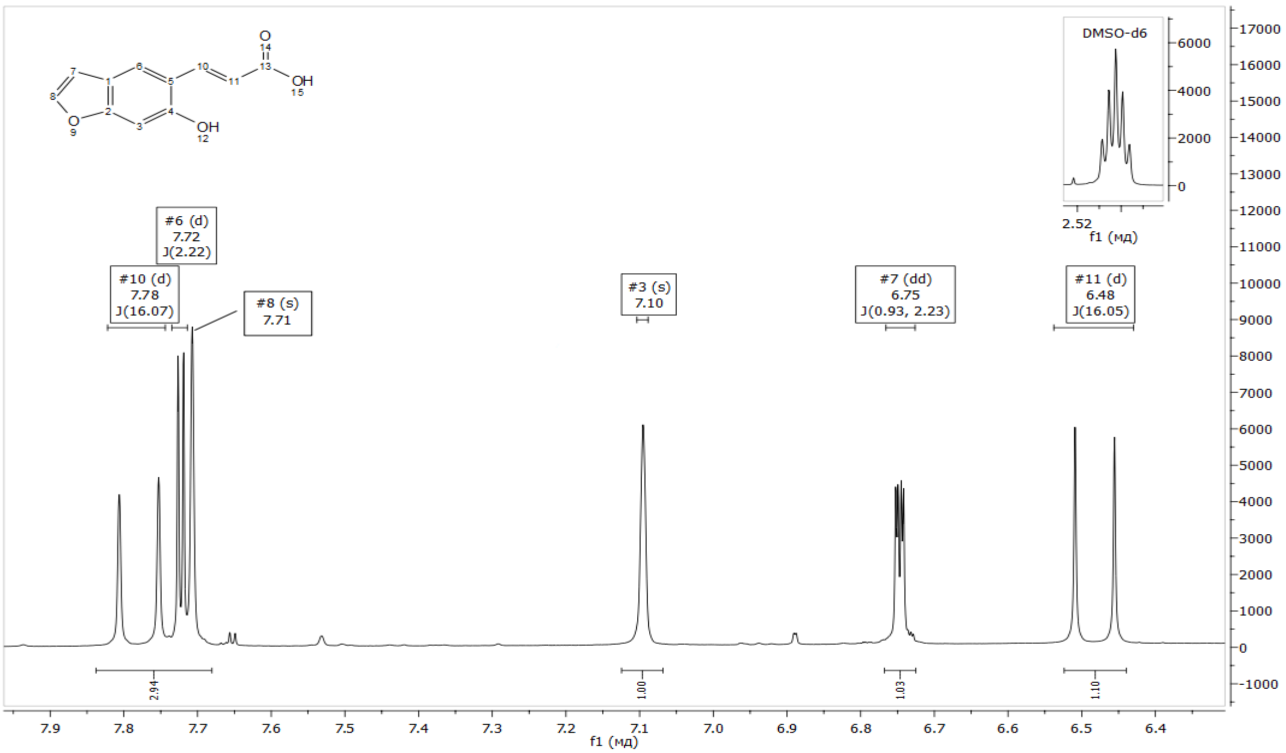

Figure S3 ¹H NMR spectrum of *E*-FCA.

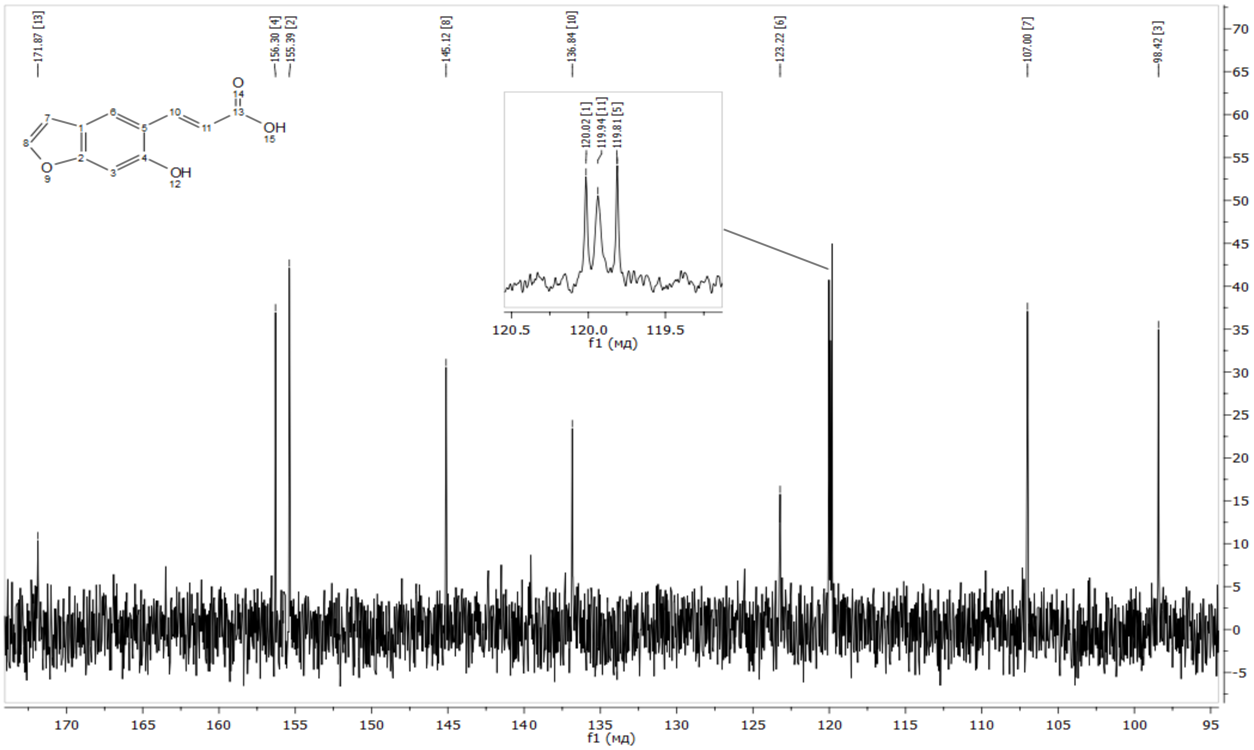

Figure S4 ¹³C NMR spectrum of *E*-FCA.

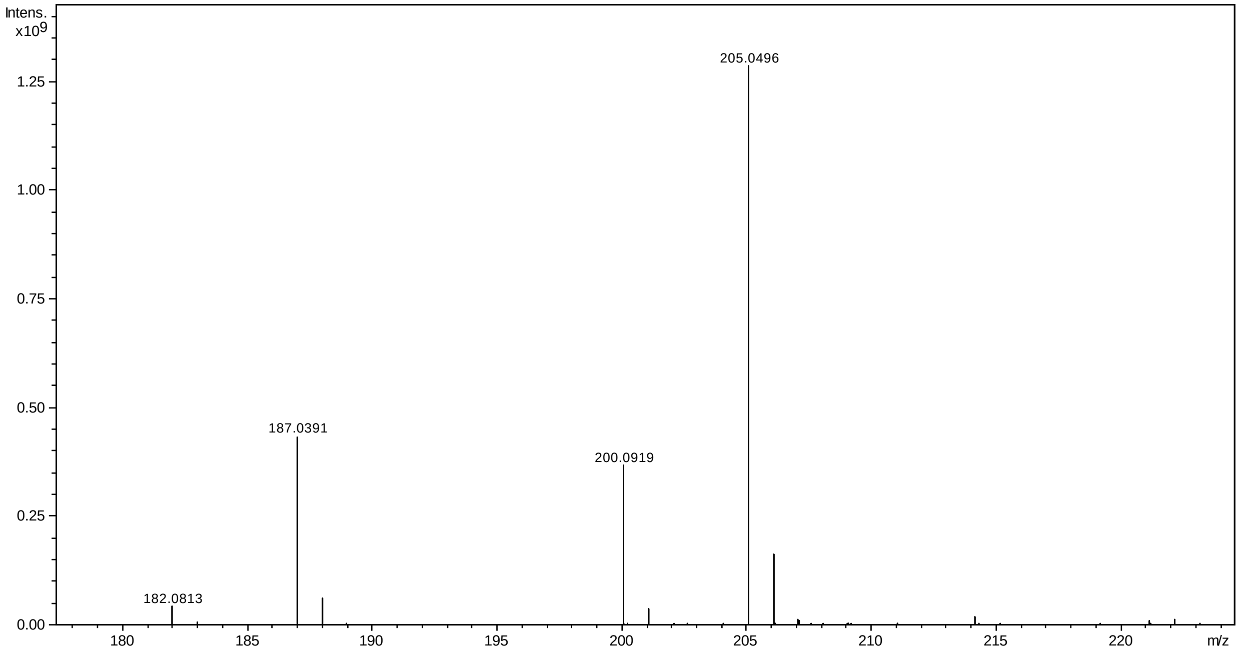

Figure S5 **HRMS spectrum of *E*-FCA recorded in positive mode.**

Solvent – acetonitrile/H_2O (50/50) + 0.1% formic acid

HRMS (HESI): *m*/*z* calc. for $[M+H]^+$: 205.0501; found: 205.0496

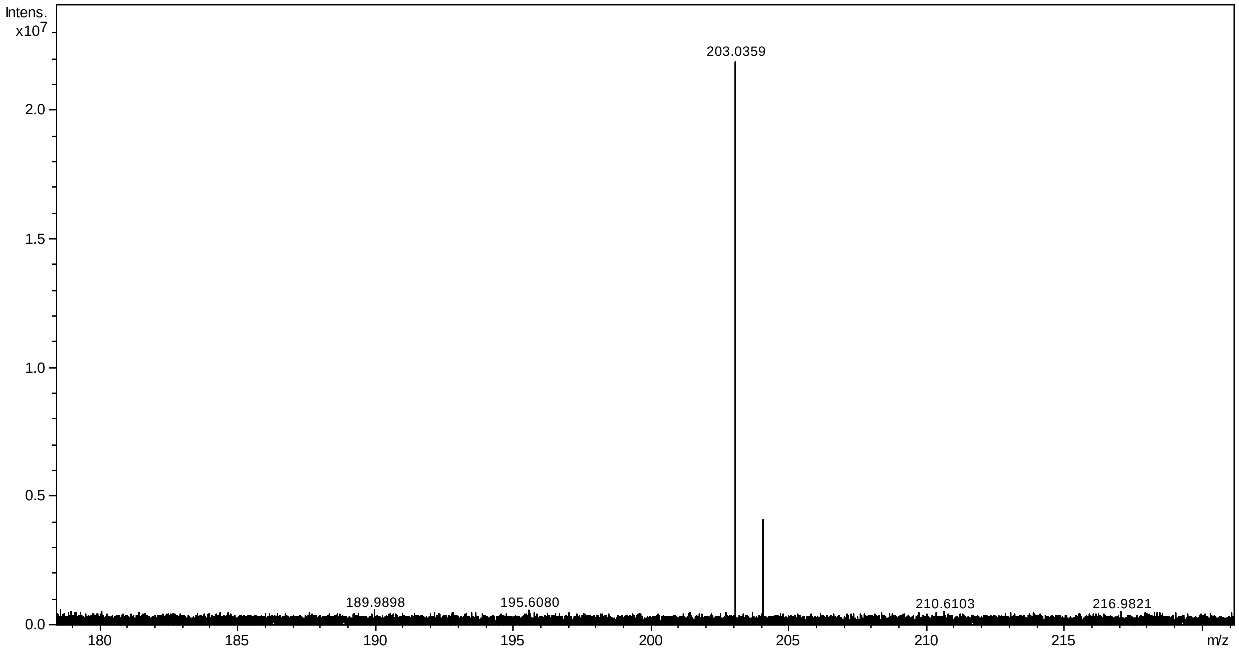

Figure S6 **HRMS spectrum of *E*-FCA recorded in negative mode.**

Solvent – MeOH

HRMS (HESI): *m*/*z* calc. for $[M-H]^-$: 203.0344; found: 203.0359

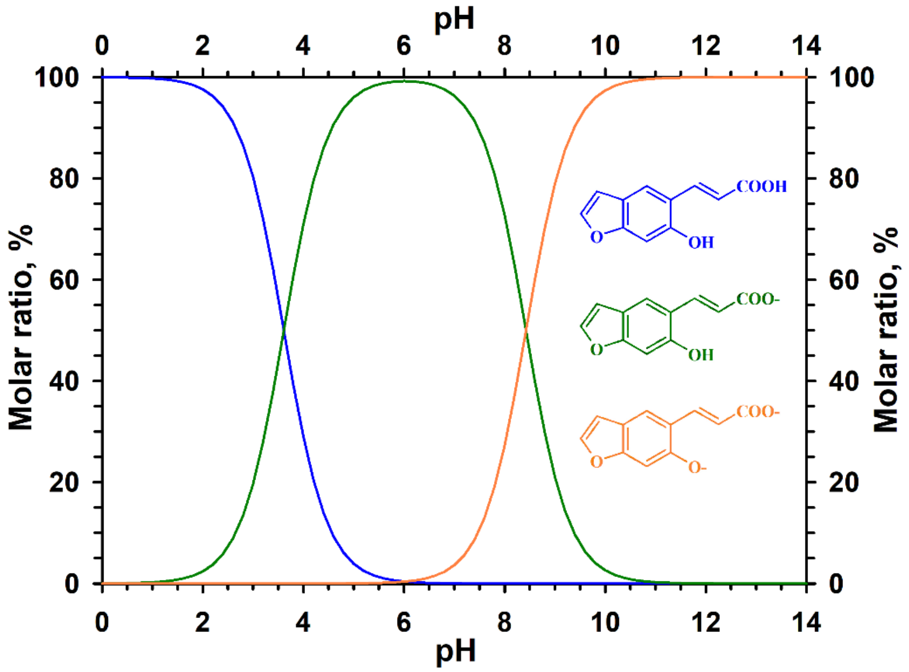

Figure S7 Calculated pH dependence of mole fractions for fully protonated, monoanionic and dianionic forms of *E*-FCA.

Theoretical pH-dependent relative distribution of fully protonated and ionized forms of ***E*-FCA** was calculated using Marvin pKa plugin (Marvin 17.6.0, ChemAxon, https://www.chemaxon.com).

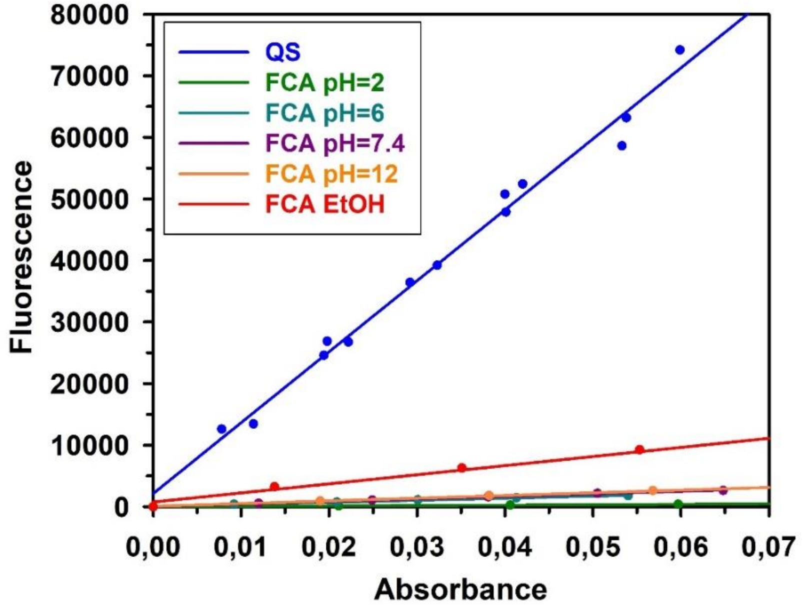

Figure S8 ***E*-FCA fluorescence quantum yield measurements.**

The relative fluorescence quantum yields for *E*-FCA at various conditions were calculated using the slope method according to the following equation:

$$\boldsymbol{\Phi_x} = \boldsymbol{\Phi_{st}} \times \frac{\boldsymbol{Slope_x}}{\boldsymbol{Slope_{st}}} \times \frac{\eta_x^2}{\eta_{st}^2}$$

where

$\boldsymbol{\Phi_x}$ is the fluorescence quantum yield of *E*-FCA; $\boldsymbol{\Phi_{st}}$ is the fluorescence quantum yield of quinine sulfate in 0.5 M H_2SO_4 ($\boldsymbol{\Phi_{st}}$ = 54.6%) used as a standard; $\boldsymbol{Slope_x}$ and $\boldsymbol{Slope_{st}}$ are the integrated fluorescence intensities *vs* absorbance for a series of dilutions of *E*-FCA and quinine sulfate, respectively, measured at the same excitation wavelength (437 nm).

To minimize the reabsorption effect, fluorescence measurements were performed in samples of *E*-FCA (12.5 µM, 0.5% of ethanol) with absorbances not exceeded 0.1 at and above the excitation wavelength.

The refractive indexes were set equal to 1.3330 for all the aqueous media and 1.3636 for 96% ethanol.

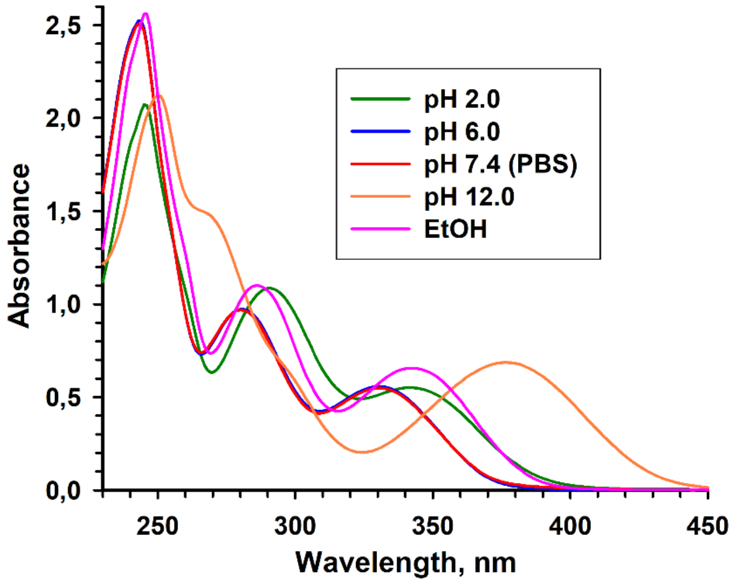

Figure S9 Absorption spectra of *E*-FCA in several solvents.

Absorption spectra of 0.1 mM *E*-FCA in several solvents were recorded in following conditions:

pH 2.0 and pH 6.0 = Aqueous solutions acidified with 1 M aq. HCl to the indicated pH values;

pH 7.4 = Solution in phosphate buffered saline (PBS);

pH 12.0 = Aqueous solution basified with 1 M aq. NaOH to the indicated pH value;

EtOH = Solution in 96% ethanol.

The solution in aqueous media contained 1% of ethanol (from stock solution).

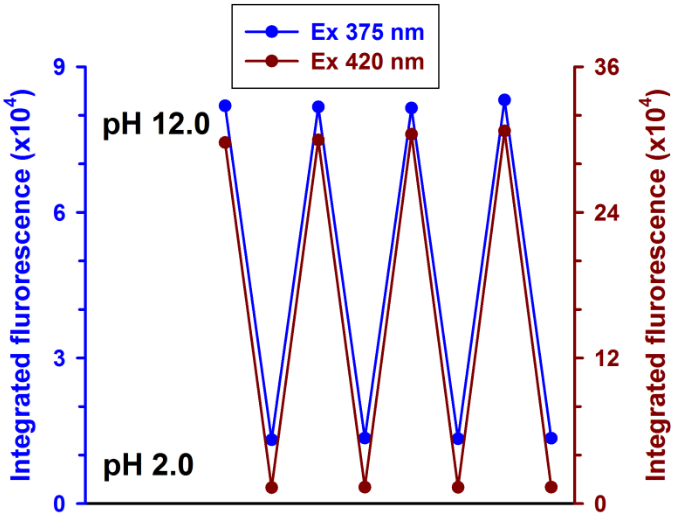

Figure S10 Fluorescence intensity recovery during cyclic changing the pH values of *E*-FCA solution (12.5 μM, 0.5% ethanol) between pH 2.0 and 12.0.

The ***E*-FCA** solutions were cyclically acidified with 1 M aq. HCl or basified with 1 M aq. NaOH to the indicated pH values, and the integrated fluorescence intensity (area under fluorescence emission curve) for each point was calculated.

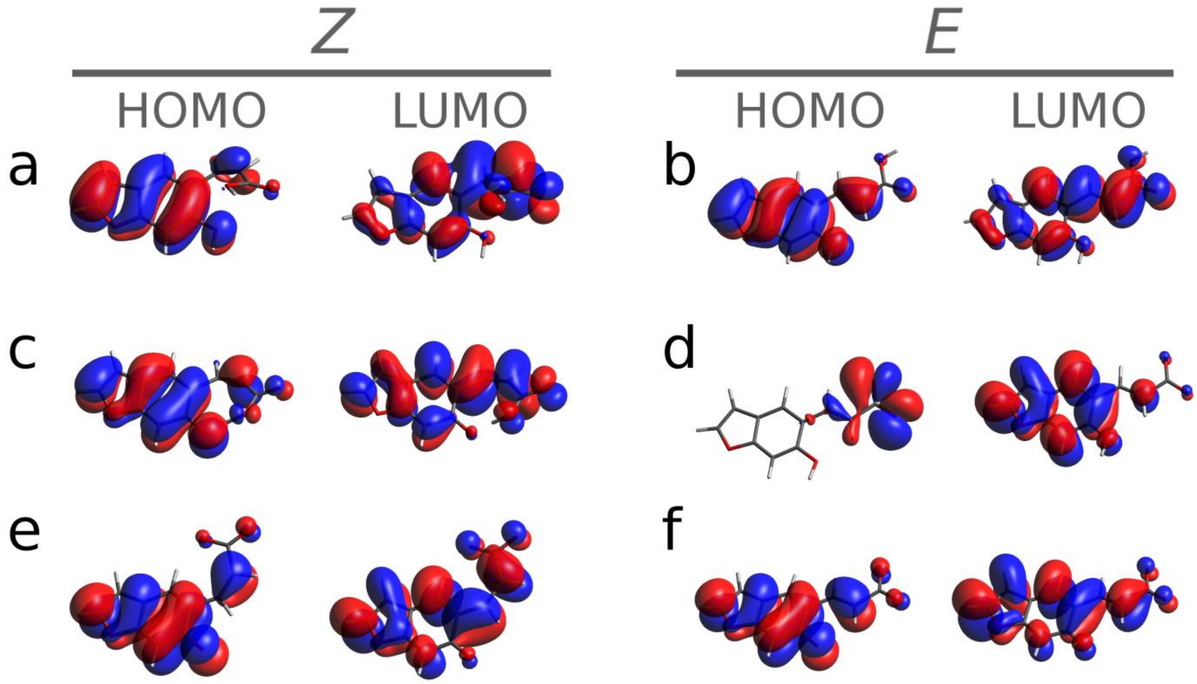

Figure. S11 The highest occupied (HOMO) and the lowest unoccupied (LUMO) molecular orbitals for protonated forms of *Z*-FCA (a) and *E*-FCA (b), monoanionic forms of *Z*-FCA (c) and *E*-FCA (d), and dianionic forms of *Z*-FCA (e) and *E*-FCA (f).

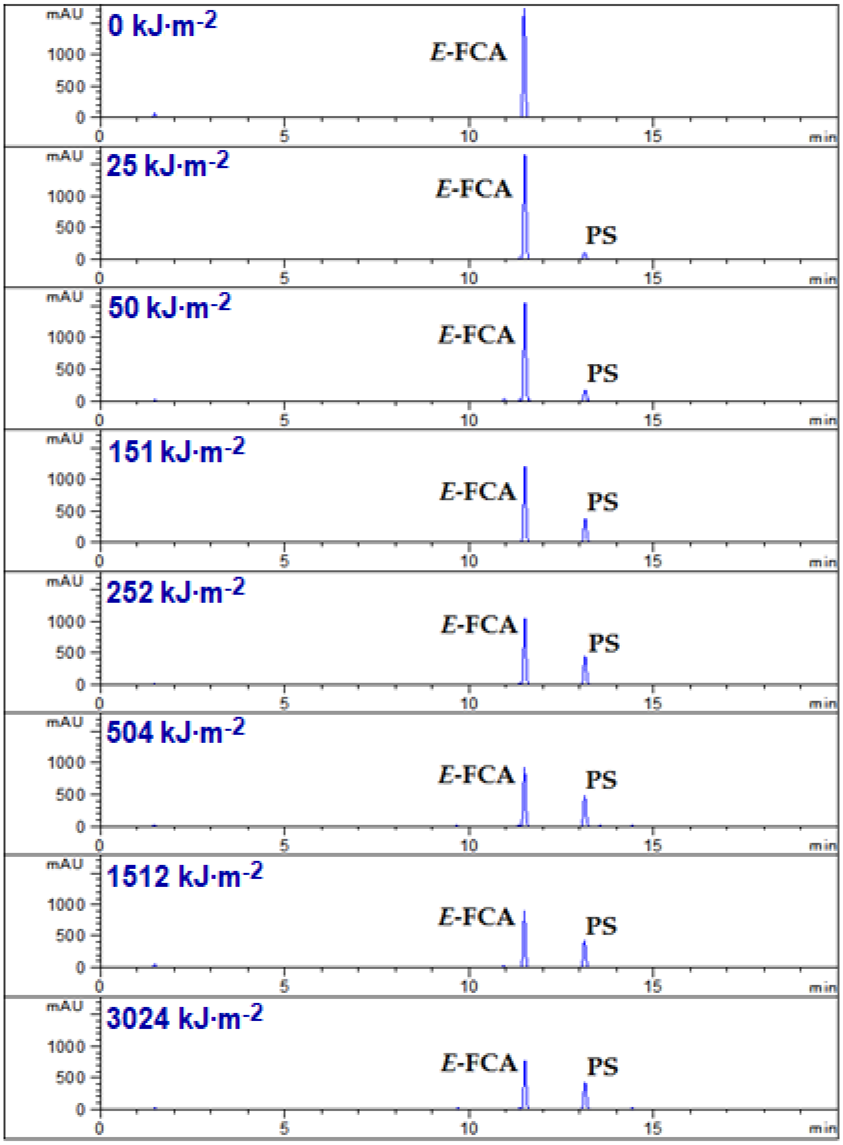

Figure S12 HPLC control of *E* → *Z* photoisomerization process.

Aqueous ***E*-FCA** solutions (0.1 mM, 1% ethanol) corrected to pH = 6.5 with 1 M aqueous HCl were UVA irradiated with high radiant flux UV LED array (365 nm, 840 W m^{-2}) with fluences 0 – 3024 kJ m^{-2} (top to bottom) corresponding to 0 – 30 min of UVA irradiation.

Prior to HPLC analysis, UVA irradiated solutions were further acidified to pH 2 with 1 M aqueous HCl.

HPLC analysis conditions: column: Agilent Zorbax® Eclipse XDB C18 (5 μm; 4,6 × 150 mm); mobile phases: A – water + 0.01% TFA, B – acetonitrile + 0.01% TFA; gradient: 2% B for 2 min, 2-82% B over 15 min, 1-min hold at 82% B, 82-2% B over 2 min; flow rate: 1.0 mL min^{-1}; temperature: 25 °C; injection volume: 100 μL; DAD detection: 254 nm.

Top chromatogram corresponds to fully protonated ***E*-FCA** (R_t = 11.5 min). On other chromatograms, left peak corresponds to ***E*-FCA**, and right peak corresponds to psoralen (**PS**, R_t = 13.1 min) as a product of ***Z*-FCA** pyrone ring back-closure.
